# Nutritional Modulation of Immune and Central Nervous System Homeostasis: The Role of Diet in Development of Neuroinflammation and Neurological Disease

**DOI:** 10.3390/nu11051076

**Published:** 2019-05-15

**Authors:** José Antonio Estrada, Irazú Contreras

**Affiliations:** Neurochemistry Laboratory, Faculty of Medicine, Universidad Autonoma del Estado de Mexico, 50000 Toluca de Lerdo, Mex., Mexico; icontrerasg@uaemex.mx

**Keywords:** diet, microbiome, immune system, neuroinflammation, neurological disease

## Abstract

The gut-microbiome-brain axis is now recognized as an essential part in the regulation of systemic metabolism and homeostasis. Accumulating evidence has demonstrated that dietary patterns can influence the development of metabolic alterations and inflammation through the effects of nutrients on a multitude of variables, including microbiome composition, release of microbial products, gastrointestinal signaling molecules, and neurotransmitters. These signaling molecules are, in turn, implicated in the regulation of the immune system, either promoting or inhibiting the production of pro-inflammatory cytokines and the expansion of specific leukocyte subpopulations, such as Th17 and Treg cells, which are relevant in the development of neuroinflammatory and neurodegenerative conditions. Metabolic diseases, like obesity and type 2 diabetes mellitus, are related to inadequate dietary patterns and promote variations in the aforementioned signaling pathways in patients with these conditions, which have been linked to alterations in neurological functions and mental health. Thus, maintenance of adequate dietary patterns should be an essential component of any strategy aiming to prevent neurological pathologies derived from systemic metabolic alterations. The present review summarizes current knowledge on the role of nutrition in the modulation of the immune system and its impact in the development of neuroinflammation and neurological disease.

## 1. Introduction

The role of diet in the development of metabolic alterations leading to chronic inflammatory disease is becoming increasingly clear, owing to extensive research on the subject in recent years. There is ample evidence connecting metabolic disease, such as obesity and and type 2 diabetes mellitus (T2DM), to dietary changes, including increased intake of high-calorie content, highly processed food and their constituent additives, as well as decreased consumption of fruits, vegetables, and other sources of dietary fiber, common characteristics of what is known as “western” diets [1,2,3].

Metabolic changes characterized by impaired glucose metabolism and insulin resistance, leading to alterations in systemic energy availability, chronic low-grade inflammatory responses, and increased oxidative stress, are hallmarks of metabolic dysregulation observed in obese and diabetic patients and are also known factors involved in development of neurological pathologies including Alzheimer’s (AD), Parkinson’s (PD), and Huntington’s (HD) diseases. High body-mass index (25–30 or above) is related to two- to three-fold increased risk for developing dementia in humans and this outcome has been attributed primarily to the effects of hyperglycemia and impaired insulin-dependent signaling, which promote inflammation and oxidative stress both systemically and in the central nervous system (CNS), leading to neuronal and glial damage and development of neurodegenerative pathologies [4]. Furthermore, chronic metabolic dysregulation increases the risk of cognitive impairment and dementia in patients with diabetes, due to enhanced glucose-dependent oxidative stress, presence of advanced glycation end products, inflammation and atrophy of brain regions like the hippocampus and amygdala [5].

Adequate nutrition is necessary to prevent metabolic dysfunction, as well as promoting immune and nervous system homeostasis and specific dietary patterns have been extensively linked to beneficial health effects. For example, Mediterranean-style and other fruit- and vegetable-rich diets, which comprise high concentrations of components with anti-oxidant or anti-inflammatory properties, are highly recommended for the prevention of metabolic and chronic-degenerative diseases [6]. However, evaluation of the effects of specific dietary patterns on health are difficult to perform, since multiple individual variables must be taken into account to understand the effects of specific dietary components on health.

There is accumulating evidence on the link between diet and mental health, in particular through the dependence of systemic metabolism on the quality of diet, but also due to the effects of dietary components on immune functions and inflammation, not only at the intestinal level, but also in other organs and tissues, including the central nervous system. The following sections will describe the modulatory effects of dietary and intestinal microbiome-derived components on the immune system and how they may relate to immune dysregulation and development of neurologic pathologies.

## 2. The Gut-Microbiome-Brain Axis

The human microbiome comprises a variety of microorganisms from multiple phyla, inhabiting the skin and mucosal tissues [7]. The intestinal microbiota, in particular, is comprised mainly by five different phyla and multiple genera of the Eubacteria domain: Actinobacteria (*Bifidobacterium*), Bacteroidetes (*Bacteroides* and *Prevotella*), Firmicutes (*Clostridium* and *Lactobacillus*), Proteobacteria (*Escherichia*), and Verrucomicrobia (*Akkermansia*), which present significant variability among individuals and populations, with some estimates suggesting over 1000 bacterial species that may be present within specific populations [8,9,10]. The relevance of the microbiome for the modulation of metabolism has become evident with studies on biochemical parameters related to glucose tolerance and insulin production in germ-free mice [11], with further evidence emerging from studies on changes in the susceptibility to obesity in genetically obese and lean animals with altered microbiomes [12]. Presently, the microbiome is known to participate in the regulation of multiple physiological processes, including energy balance and the development and functions of the immune system, with alterations in its composition and functions modifying the development and evolution of multiple pathologies [13].

The microbiome in the gastrointestinal system is an essential component of systemic metabolism, as the bacteria it contains plays an important role in nutrient absorption and synthesis of multiple metabolites relevant for human health, including lipids, amino acids, vitamins, bile acids, and short-chain fatty acids (SCFAs), as well as bacteria-specific products including peptidoglycans and lipopolysaccharides [10,14]. The role of the gut microbiome in energy metabolism in humans is still being elucidated. It has been suggested that specific bacterial genera are responsive to energy availability in the organism or to the calorie content in diet, as obese mice and humans present an increased amounts of Firmicutes and decreased amounts of Bacteroidetes in the intestine, and these levels are reversed when the animals are fed a calorie-restricted diet or when weight is lost, either by reducing calorie intake or by surgical procedures [15,16,17,18]. In addition, germ-free mice that are reconstituted with bacteria from obese animals gain more weight and adipose tissue than those reconstituted with bacteria from lean animals, suggesting that the intestinal microbiome is relevant for regulation of energy metabolism in the host [12].

In addition to its regulatory effects on metabolism, the intestinal microbiome plays an essential role in the modulation of immune and nervous system functions, either through direct production of bioactive components affecting the release of hormones, incretins and neurotransmitters, or by regulation of leukocyte functions, such as cytokine production [19]. Therefore, the microbiome is part of a complex bidirectional communication system integrating the gastrointestinal, immune, and nervous systems and its stability and adequate functional status is necessary for maintenance of human health.

## 3. Dietary Patterns and Regulation of the Microbiome

Diet has been extensively demonstrated to modify the colonization and permanence of intestinal bacterial populations. In general, studies point to specific “enterotypes” in humans, which are determined by the presence of specific bacterial sub-types that can be found in different populations [20,21]. These enterotypes are shaped in large part by the particular long-term dietary habits of every individual; for example, protein- and fat-rich diets favor the proliferation of bacteria of the *Bacteroides* genus [22]. Defining dietary patterns is a complex issue, since they vary widely, even within a specific geographic region. Therefore, these patterns are usually defined based on a particular set of dietary components that are prevalent in each region and can be found in increased quantities in each diet sub-type, even though each pattern may comprise multiple sub-regional variations with different ingredients and amounts used, but which maintain the general characteristics of a given pattern. The following sections illustrate the kinds of components that characterize each specific pattern described and their known effects on microbiome composition.

### 3.1. Western-Type Diets and the Intestinal Microbiome

Western-type diets are usually considered to have high concentrations of dietary fat, consisting particularly of saturated fatty acids (e.g., butyric, lauric, myristic, palmitic, and stearic acids), which are non-essential lipids found in large quantities in animal products, containing no double carbon-carbon bonds and related to increases in blood triglycerides and cholesterol levels, as well as very high quantities of red meat protein, sugar, salt, and low amounts of dietary fiber, due to low consumption of fruits and vegetables [23]. The high-carbohydrate, high-fat, low-fiber characteristics of western-type diets have profound effects on the composition of the intestinal microbiome and the regulation of the immune system, as they are related to an enhanced pro-inflammatory intestinal milieu and development of metabolic and immune abnormalities that are relevant for the incidence and evolution of chronic and degenerative pathologies. Western-type diets favor the proliferation of gram-negative bacteria like *E. coli*, which produce endotoxins and contribute to systemic pro-inflammatory immune responses and metabolic dysregulation [24,25].

### 3.2. Mediterranean Diets and the Intestinal Microbiome

In contrast to western-type diets, traditional Mediterranean diets are characterized by high intake of low-starch vegetables, fruits, legumes, nuts, and whole-grain cereals, using olive oil as a main source of unsaturated fat. This fiber-rich diet has been related to several health benefits, including lower blood cholesterol, inflammation, and oxidative stress [26]. Mediterranean diets usually contain high quantities of unsaturated fatty acids (e.g., linolenic, linoleic, eicosapetaenoic, docoshexaenoic, and arachidonic acids), essential components of cellular membranes which contain at least one double carbon-carbon bond and which include the omega-3 and omega-6 fatty acids found in large quatities in fish and nuts, as well as low glycemic index carbohydrates, which promote healthy metabolic profiles in humans [27]. In contrast to western-type, protein-rich diets, the low concentrations of animal protein and fat, along with increased quantities of complex carbohydrates and fiber in Mediterranean diets, promote the proliferation of *Bacteroides*, *Prevotella*, and *Faecalibacterium* species, which are considered beneficial for health, while decreasing the proportion of potentially detrimental Firmicutes and Proteobacteria [28,29].

### 3.3. Asian Diets and the Intestinal Microbiome

Asian-style diets are typically rich in polysaccharides from cereals, particularly rice, as well as different root vegetables, like onion, garlic and ginger, as well as algae, along with protein and fat derived from fish and soy. Traditional Asian diets present high concentrations of quercetin and alliin, plant-derived components with anti-inflammatory and anti-oxidant functions that relate to increased presence of Firmicutes genera, with decreased presence of Bacteroidetes and Proteobacteria in the intestine, concomitant with reduced incidence of inflammatory bowel disease [30,31]. Microbial production of immunoregulatory SCFAs, including butyrate, acetate, and indole, are also increased in animals fed Asian-style diets [30].

The general effects of dietary fiber, polysaccharides, fat, and proteins, found in different proportions in these diets, on the intestinal microbiome, are described below.

### 3.4. Effect of the Main Groups of Dietary Components on the Microbiome

#### 3.4.1. Dietary Fiber and Polysaccharides

Significant modifications to intestinal bacterial populations may be observed within two days of acute dietary modifications, such as going from low-fiber to high-fiber or increasing/decreasing the amount of meat-derived protein in diet, even though the general composition of the microbiome is fairly stable and dependent on long-term dietary habits [22,32]. The microbiome plays important roles in the metabolism of dietary components within the intestine; fiber is particularly important in shaping the microbial composition in the colon, which harbors the highest density of microbes within the intestine [33]. Bacteria utilize the complex carbohydrates found in dietary fiber to produce a variety of SCFAs, including acetate, propionate, and butyrate, which are relevant for energy metabolism within the host, as well as for regulation of intestinal pH, which is in turn important for the presence of specific bacterial genera in the intestine [11,34].

The intestinal microbiome is very sensitive to the specific components found in different types of dietary fiber, producing variable amounts of SCFAs depending on its composition [35]. Increased concentrations of fermentable fiber in diet leads to increased production of SCFAs in the colon that are absorbed in part by exchange with bicarbonate, modifying the colonic pH throughout the distal colon [36]. Variable pH levels affect the composition of the local microbiome and play a role in preventing the growth of pathogenic bacteria, with oligosaccharide-rich diets promoting growth of anaerobic bacteria and higher SCFAs concentrations, compared to cellulose, in animals [36]. Furthermore, dietary fiber-dependent SCFA production regulates glycaemia by stimulating production of the gastrointestinal hormones tyrosine-tyrosine peptide (PYY), which induces satiety and enhances insulin sensitivity, as well as glucagon-like peptide 1 (GLP-1), which promotes pancreatic insulin secretion, with the overall effect of enhancing glucose tolerance [37,38,39]. Additionally, SCFAs have also been shown to decrease hepatic cholesterol synthesis [40]. Importantly, germ-free animals produce diminished concentrations of SCFAs and have decreased adipose tissue mass, even when fed with carbohydrate-rich diets, which can be normalized when the microbiome is reconstituted [11]. Similarly, diets enriched in plant polysaccharides favor the proliferation of Bacteroidetes, while decreasing that of Firmicutes, since bacteria from the former phylum contain bacteria such as *Prevotella* that is capable of metabolizing cellulose [41].

Galacto-oligosaccharides and Fructo-oligosaccharides are found in human milk and have an important role in establishing the microbiome in infants early in development. These oligosaccharides, which are produced by enzymatic modification of lactose and inulin, respectively, promote the growth of *Bifidobacteria* and *Lactobacilli* [42]. These bacteria are relevant in the maturation of the intestinal epithelium, as well as in the establishment of the intestinal immune system and the regulation of leukocyte proliferation and secretion of cytokines like IFN-γ and IL-10 by leukocytes in circulation [43].

#### 3.4.2. Dietary Fat

The quantity and quality of dietary fat is also important to determine intestinal colonization and proliferation of specific bacterial species, although its effects do not appear to be as pronounced as those from dietary fiber and polysaccharides. There are reports demonstrating the effects of low- and high-fat diets on the intestinal microbiome, increasing the proportion of *Faecalibacterium* and *Bacteroides*, respectively [44]. Similarly, high-fat diets have been shown to rapidly affect the composition of the intestinal microbiome, favoring the proliferation of Firmicutes and Proteobacteria over Bacteroidetes [45]. These alterations are also related to increased concentrations of lipopolysaccharides in circulation in animals and humans fed with fat-rich diets [46,47]. However, further evidence shows that polyunsaturated fatty acids do not have a clear effect on the intestinal microbiome, whereas diets rich in monounsaturated fatty acids decrease the total amount of intestinal bacteria and high-fat diets rich in saturated fatty acids limit the diversity of the intestinal microbiome [48].

#### 3.4.3. Protein

In contrast to lipids, the protein content in diet has important implications for the metabolic profile of the intestinal microbiome. High-protein diets are directly related to proliferation of *Escherichia* species and with decreased presence of *Bifidobacterium*, *Prevotella*, and *Akkermansia* species in animal models [23]. Studies in humans suggest that high-protein diets do not have a large impact on the composition of the intestinal microbiome, but that they modify bacterial amino acid metabolism, producing metabolites with multiple modulatory functions, depending on the type of proteins found in diet [49]. These metabolic alterations are related to increased risk for intestinal pathologies, since they reduce the production of immunoregulatory specific SCFAs, like butyrate, as well as the concentration of anti-oxidant compounds [49].

## 4. Microbiome-Dependent Regulation of the Immune and Nervous systems

**Figure 1:**
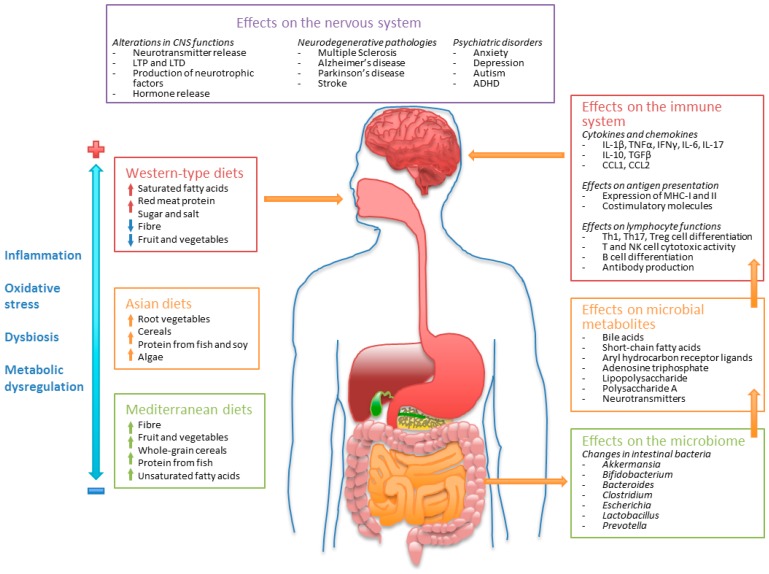
Dietary patterns and modulation of the intestinal microbiome, immune, and nervous systems. Dietary patterns promote changes in the intestinal microbiome, affecting the production of bacterial metabolites that, in turn, modulate immune and nervous system functions, either increasing or decreasing the susceptibility for neurologic and psychiatric disorders. Western-style diets are related to increased inflammation, oxidative stress, dysbiosis, and metabolic abnormalities, whereas traditional Mediterranean and Asian diets are considered beneficial for human health [24,25,26,27,28,29,30,31]. CNS: Central nervous system; LTP: Long-term potentiation; LTD: Long-term depression; ADHD: Attention deficit hyperactivity disorder.

Once established, the intestinal microbiome becomes a permanent source of bioactive metabolites with multiple regulatory functions on systemic metabolism. Metabolites produced by the intestinal microbiome can modulate immune and nervous system functions through multiple mechanisms. The modulatory effects of some of the most well studied microbiome-related metabolites on these systems are described below and are shown in Figure 1.

### 4.1. Bile Acids

Extensive evidence has linked the development of inflammatory bowel disease (IBD) to disruption of the intestinal microbiome and decreased concentrations of secondary bile acids. Bacteria in the intestine are capable of converting primary bile acids into secondary bile acids by dehydration and deconjugation of the aminoacids taurine and glycine from their structure, after they have been modified in the liver, thus changing their properties and functions and regulating bile acid excretion [14,50]. Both primary and secondary bile acids are considered modulators of leukocyte functions through the activation of G protein-coupled bile acid receptor 1 (GPBAR1) and nuclear receptor subfamily 1 H4 (NR1H4; also known as farnesoid X-activated receptor, FXR) [51]. Both receptors are expressed in different leukocyte subpopulations and have been linked with anti-inflammatory activity dependent on downregulation of STAT-1 and NF-κB signaling in macrophages and dendritic cells, decreasing production of nitric oxide and the pro-inflammatory cytokines IL-1β, TNF-α, and IL-6 [52,53,54]. Absence of GPBAR1 in knockout animals worsens pathology in models of intestinal inflammation [55].

The relevance of bile acids for the modulation of neurological disease pathology has been observed in animals and humans, as altered concentrations of bile acids are observed in the cerebrospinal fluid of patients with hepatic encephalopathy and neurodegenerative diseases like multiple sclerosis, as well as in the brain of animals in the experimental autoimmune encephalomyelitis (EAE) model [56,57,58]. There is evidence that both primary and secondary bile acids are synthesized within the brain [59] and they are capable of inhibiting glutamatergic and gabaergic signaling through FXR, affecting respiratory activity, motor coordination, and memory processes in FXR-knockout mice [59,60,61]. In addition, increased circulatory bile acid concentrations, such as those observed in experimental obstruction of biliary ducts, promote an increase in blood-brain barrier permeability, and the accumulation of bile acids within brain structures like the hypothalamus, where they interfere with production of hormones like corticotrophin-releasing hormone, leading to alterations in the hypothalamus-pituitary-adrenal (HPA) axis and the stress response [62,63,64,65].

The possibility of using bile acids as a therapeutic alternative for the treatment of neurological disease stems from studies demonstrating that signaling through bile acid receptors have anti-inflammatory roles in the EAE model, reducing disease severity [66]. Additionally, dietary supplementation with conjugated bile acids has been shown to ameliorate pathology in animal models of AD and PD [67,68].

### 4.2. Short-Chain Fatty Acids (SCFAs)

SCFAs are a group of saturated organic acids, containing one to six carbon atoms, with acetate, propionate, and butyrate being the most common in the human intestine. Dietary fiber is the common substrate for the production of SCFAs in the gastrointestinal system. Humans are incapable of digesting most of the structural carbohydrates found in dietary fiber, such as cellulose and inulin, which traverse the intestinal tract until they reach the cecum and colon, were they are fermented by bacteria, producing SCFAs as a metabolic byproduct [69]. It is estimated that SCFAs account for around 10% of daily caloric production from diet in humans, with butyrate acting as the main source of energy for enteric cells in the colon, while the liver is able to uptake circulating SCFAs to promote synthesis of fatty acids, cholesterol, glucose, and other metabolic products [36,70,71,72]. Absorbed SCFAs interact with cellular components through G protein-coupled receptors, particularly GPR43 (also known as free fatty acid receptor 2, Ffar2) and GPR41 (Ffar3) [73]. These receptors are highly expressed in different leukocyte subpopulations [73]. SCFAs have potential for the prevention and treatment of metabolic and inflammatory pathologies like metabolic syndrome, intestinal bowel disease, ulcerative colitis, Crohn’s disease, and colon cancer, as they present potent immunomodulatory activity [74,75,76,77].

Signaling through GPCRs activates multiple cascades that are relevant for leucocyte activation and effector functions, including mitogen-activated protein kinases (MAPKs) ERK 1/2, JNK, and p38 [78], as well as regulating DNA transcription by inactivating histone deacetylases [79]. In addition, SCFAs entering the cell can activate the peroxisome proliferator-activated receptor gamma (PPARγ), which may promote anti-inflammatory effects under specific conditions [80]. Binding of SCFAs to GPCRs enhances neutrophil chemotaxis, as well as proliferation of macrophages and dendritic cells, while also inhibiting pro-inflammatory cytokine production [81,82,83]. SCFAs inhibit the maturation of dendritic cells and enhance Th2 polarization by decreasing production of IL-12 and IFN-γ and expression of MHC-II and the costimulatory molecules CD80, CD86, and CD40, while increasing production of IL-10 and IL-23, in response to butyrate [84,85]. Butyrate has also been shown to inhibit proliferation and enhance apoptosis of T cells [86]. Decreased Th2 responses have been directly linked to the fiber content in diet, as high-fiber diets decrease allergic airway inflammation in murine models by decreasing Th2-mediated IgE antibody production [87].

Additionally, SCFA-dependent signaling through GPR43 promotes chemotaxis, phagocytic activity, and production of reactive oxygen species in neutrophils [88], as well as decreasing production of the macrophage chemotactic protein CCL2 and the cytokines TNF-α, IFN-γ, and IL-10 by blood-derived mononuclear cells, whereas knocking out GPR43 enhances intestinal inflammation by promoting production of TNF-α and IL-17 [89,90]. Along with GPR43, an additional receptor for SCFAs, GPR109A, has been shown to modulate inflammasome activation and production of IL-18, a relevant cytokine for the maintenance of intestinal barrier integrity, and its functions are dependent on the fiber content in diet and on intestinal dysbiosis [91]. Furthermore, both butyrate and propionate have been shown to enhance peripheral differentiation and proliferation of Treg cells, providing additional evidence for the immunomodulatory role of SCFAs in vivo [92].

SCFAs have also been related to development or progression of neurological pathologies. There are reports on decreased production of microbiome-derived SCFAs in patients with multiple sclerosis and several studies have demonstrated anti-inflammatory properties for these molecules in the EAE model [93]. These regulatory properties are thought to derive from inhibition of Th1 and Th17 responses and promotion of Treg differentiation and production of anti-inflammatory cytokines [94]. Similarly, microbiome-dependent SCFA production is reduced in animal models of AD and these molecules are able to prevent clumping of amyloid beta peptides into neurotoxic aggregates in vitro, although their therapeutic potential in humans has not been determined [95]. Microbiome-derived SCFAs also have a permanent role in the modulation of microglial phenotype and effector functions. Germ-free or antibiotic-treated mice present microglial alterations characterized by decreased expression of genes related to JAK/STAT signaling pathways and reduced expression of MHC-I and costimulatory molecules, appearing to have an immature phenotype, which can be counteracted by reconstitution of the microbiome. These alterations are dependent on expression of GCPR43 and production of SCFAs and may have important implications for microglial functions in health and disease [96].

### 4.3. Aryl Hydrocarbon Receptor Ligands

The aryl hydrocarbon receptor (AhR) is a nuclear receptor recognized for its involvement in the detoxification of xenobiotics through cytochrome enzymes and is known to respond to tryptophan- and microbiome-derived metabolites, including SCFAs, as well as several endogenous molecules, like bilirubin, biliverdin, arachidonic acid metabolites, including prostaglandins, leukotrienes, and lipoxins, as well as kinurenines, serotonin, and triptamine, derived from endogenous and microbiome-dependent tryptophan metabolism [97,98,99]. It presents high expression in epithelia and appears to be necessary for adequate development of the gastrointestinal immune system and intestinal epithelial barrier [100].

Signaling through the AhR is required for the development of intestinal intraepithelial lymphocytes [101] and it has been linked to the microbiome-dependent inhibition of colitis [102]. The AhR has been shown to have a crucial role in the regulation of pro-inflammatory responses activated by bacterial antigens, particularly lipopolysaccharide (LPS), since AhR deficiency in knockout mice promotes increased production of IL-1β, IL-6, IL-12, TNF-α, and IFN-γ after inoculation with LPS, promoting the development of septic shock [103]. The anti-inflammatory activity of AhR is dependent on the production of kynurenines, which are synthesized from tryptophan by indoleamine dioxygenase (IDO) enzymes 1 and 2 in response to antigenic stimuli in leukocytes [104]. The kynurenines signal through the AhR to inhibit expression of pro-inflammatory cytokines and control long-term tolerance to specific bacterial antigens [105]. Activation of the AhR also regulates proliferation of pro-inflammatory Th17 leukocyte subpopulations and promotes anti-inflammatory IL-10 and IL-22 production, macrophage survival, and production of reactive oxygen species in phagocytes [106,107,108]. IL-22 is particularly relevant for the maintenance of the integrity and homeostasis of the intestinal epithelium and is mainly produced by type 3 innate lymphoid cells in an AhR-dependent manner [109].

In addition to its role in the regulation of inflammatory processes, the AhR is necessary for proper myelination of neuronal axons in the CNS. AhR deficiency has been demonstrated to affect the expression of myelin-associated glycoprotein in the developing optic nerve in knockout mice, leading to alterations in the processing of visual stimuli and development of nystagmus [110]. Changes in myelination in AhR-knockout mice are also accompanied by increased inflammation in the optic nerve, evidenced by astrogliosis and enhanced expression of IL-1β, TNF-α, CCL1, CCL5, and CCL7 [110]. Similarly, AhR has been shown to participate in the development of mature neuronal synapses in the hippocampus and in neurogenesis of cerebellar granule neurons in AhR-deficient mice [111].

Further evidence for the role of AhR in the regulation of CNS inflammation comes from studies showing decreased production of AhR ligands in relapsing-remitting MS patients and upregulation of these ligands during active disease [112], as well as the demonstration of the relevance of type I interferon-dependent signaling through AhR in astrocytes to reduce inflammation and disease severity in the EAE model [113]. Furthermore, AhR signaling appears to be at least partially dependent on tryptophan metabolism by the microbiome in this model, as antibiotic treatment enhances disease severity, which can be inhibited by supplementation with tryptophan metabolites [113].

### 4.4. Adenosine Triphosphate

Intestinal bacterial are capable of releasing ATP into the intestinal milieu [114]. Extracellular ATP functions as a signaling molecule with relevant pro-inflammatory properties, which is also released from damaged cells in all tissues, as well as by activated leukocytes [115]. Bacterial ATP, signaling through purinergic P2X or P2Y receptors, promotes chemotaxis and pro-inflammatory cytokine production by leukocytes, enhancing production of CCL2, CCL3, and CXCL8, along with IL-6, TNF-α, IL23p19, and TGF-β by intestinal dendritic cells, as well as promoting differentiation and proliferation of Th17 cells [114,116]. These effects are reduced in germ-free mice and can be rescued by intestinal or systemic administration of exogenous ATP [117]. Additionally, activation of P2X receptors also promotes production of IL-1β and IL-18 by antigen-stimulated macrophages [118], as well as enhancing intestinal B cell activation and secretion of IgA and regulating lymphocyte apoptosis [119].

In contrast, enzymatic hydrolysis of ATP to adenosine promotes anti-inflammatory effects by signaling through adenosine P1 receptors, particularly A2A and A2B, whose expression is upregulated upon activation in most leukocytes [120]. Activation of A2 receptors in dendritic cells downregulates the expression of MHC-II and costimulatory molecules and the production of CCL2, CCL12, CXCL10, IL-12, and TNF-α [121], while enhancing expression of IL-6, IL-10, and TGF-β, suggesting the induction of a tolerogenic phenotype [122,123]. In T cells, adenosine modulates TCR-dependent activation signals and A2A receptors are important for T cell maturation and survival, as well as for inhibiting production of IL-2, TNF-α, IFN-γ, and CD8+ T cell-mediated cytotoxic responses [124,125,126]. Signaling via A2A also enhances Treg proliferation and expression of the inhibitory molecule PD-1 [127].

Ionotropic P2X and metabotropic P2Y receptors are also expressed in astrocytes, oligodendrocytes and microglia in the CNS, with the possibility of neuronal expression still in question. ATP is an important CNS signaling molecule, regulating neurotransmission, and myelination at neuronal synapses [128,129]. P2X receptors have been related to inflammatory post-ischemic damage in animal models of stroke and hippocampal ischemia-reperfusion injury, where inhibition of P2X7 receptors promotes neuronal survival and maintains cognitive performance [130]. Inhibition of these receptors also inhibits epileptic activity in kainate-induced epilepsy models [131]. P2X receptors are also associated with release of IL-1β in the peripheral nervous system in neuropathic pain and activation of these receptors in microglia is considered a common characteristic in neurodegenerative diseases, including multiple sclerosis, amyotrophic lateral sclerosis, AD, PD, and HD [132]. The inflammatory processes that underlie pathology in neurodegenerative disease promote cellular release of ATP, which is then free to signal through P2X and P2Y receptors to modulate cellular functions. Alterations in ATP receptor expression has been found in animal models and patients with neurodegenerative disease, and blocking signaling through P2X7 receptors can prevent neuronal and oligodendrocyte apoptosis in experimental models of neurological pathology [132].

### 4.5. Polysaccharide A and Lipopolysaccharide

Microorganisms of the *Bacteroides* genus produce the sugar polymer polysaccharide A as part of their capsules [133]. This microbial product is capable of polarizing the adaptive immune response toward the Th2 and Treg phenotype by promoting dendritic cell activation and maturation via TLR2 signaling and stimulating secretion of the anti-inflammatory cytokine IL-10 by antigen-specific CD4+ T cells, while enhancing phagocytosis and nitric oxide production by macrophages [134,135]. In the nervous system, polysaccharide A provides protection against demyelinating disease by enhancing CD4+ T cell differentiation into Treg cells and increasing production of IL-10 [136]. The beneficial effects of polysaccharide A on EAE appear to depend on CD4+ T cell expression of CD39 [137].

In contrast, lipopolysaccharide (LPS) is produced by proteobacteria like *Escherichia coli* and is a classical activator of pro-inflammatory responses by innate immune cells. It can be found in low concentrations in human plasma [47]. It has been shown to modulate macrophage infiltration into adipose tissue in mice, an effect that is related to metabolic dysregulation in obesity [138]. Intestinal LPS is also involved in development of liver insulin resistance and adipose tissue-dependent inflammation, and its plasma concentrations are increased in patients with T2DM [46]. Importantly, high-fat dietary regimes increase the amount of LPS-producing intestinal bacteria, as well as plasma LPS concentrations, in both animals and humans [139]. Development of liver and adipose tissue insulin resistance has been related to LPS-dependent activation of Toll-like receptor 4 (TLR4) in macrophages, independently of obesity [140].

LPS is known to activate M1 phenotypes and pro-inflammatory functions in phagocytic cells, including microglia, after acute stimulation. In accordance with this effect, intranasal administration of LPS is used to promote neuroinflammation and dopaminergic neuron loss in experimental animal models of PD, and disease severity increases with age, demonstrating a potential role of this bacterial product in the induction of neuroinflammation and neurological pathology in vivo [141].

Although a pro-inflammatory phenotype for glial cells is usually considered detrimental in neurological disease, activation of phagocytic activity promotes indirect neuroprotective effects in transgenic mice engineered to induce amyloid beta accumulation in the brain, as LPS inoculation promotes clearing of amyloid beta deposits by microglia, leading to decreased production of IL-1β, TNF-α, and CXCL1, thus limiting the inflammatory response in the brain. This effect is thought to depend on enhancement of microglial phagocytosis of amyloid plaques by LPS-dependent activation of TLR4 [140]. However, microglia responses to LPS decrease with age in these transgenic animals, suggesting a mechanism for disease progression related to impaired microglial phagocytosis of amyloid beta plaques in aged animals [142,143].

In contrast, LPS-stimulation may promote microglial polarization toward an M2 phenotype by indirect mechanisms, as it has been demonstrated that in vitro stimulation of macrophage cell lines with LPS promotes the release of exosomes that in turn, induce microglia polarization towards an M2 phenotype, reducing nervous tissue damage and improving neurological function in animal models of cerebral ischemia [144].

### 4.6. Neurotransmitters

Importantly, it has been demonstrated that intestinal microbes from the *Lactobacillus*, *Lactococcus*, *Bifidobacterium*, *Clostridium*, *Escherichia*, *Bacillus*, and *Saccharomyces* genii are capable of direct production of various neurotransmitters, including gamma-aminobutyric acid (GABA), serotonin, dopamine, acetylcholine, and norepinephrine, while also intervening the regulation of neurotransmitter synthesis within their host by metabolizing substrates required for these processes, like tryptophan [145].

However, there is very limited information regarding the direct modulation of inflammation and neurologic disease by bacteria-derived neurotransmitters. Most of the effects of the microbiome on neurotransmission are usually ascribed to indirect alteration of the intestinal metabolism, immune, endocrine and enteric nervous systems, leading to changes in the expression and secretion of cytokines, hormones, incretins, and neurotransmitters that affect feeding behavior and energy metabolism, including leptin, glutamate serotonin and GABA. In this way, the microbiome regulates neurotransmitter availability in the CNS and modulates neurologic functions in large part via the HPA axis and the vagus nerve [146].

Alterations in the composition or functions of the intestinal microbiome have been related to modified neurotransmission in the hypothalamus and hippocampus, promoting development of anxiety and schizophrenia [147,148], and it has also been shown that some species of Proteobacteria are capable of producing amyloid peptides, which along with polysaccharides, may promote amyloid accumulation and inflammation in the brain, becoming a potential risk factor for development of AD [149]. Therefore, recent studies have focused on the therapeutic potential of intestinal bacteria for the regulation of neurotransmitter balance in the CNS and it has been demonstrated that administration of specific bacterial strains may reverse neuroendocrine alterations involved in anxiety- and depressive-like behaviors in obese animals [150].

## 5. Dietary Patterns and Regulation of the Immune System

The composition and quality of dietary components are important determinants for adequate functioning of systemic metabolism and, therefore, have relevant roles in the regulation of all systems within the organism, including the immune system. High-fat, low-fiber western-type diets are associated to development of obesity, which is a potent promoter of systemic inflammatory responses. In contrast to adipose tissue in lean people, adipocytes in obese individuals secrete pro-inflammatory cytokines, like IL-1β, IL-6, and TNF-α, which promote inflammation in acute settings, whereas in chronic settings they promote metabolic dysregulation, insulin resistance, and overall downregulation of immune responsiveness [151]. These changes lead, among other things, to increased susceptibility to infection and development of metabolic syndrome and T2DM in obese individuals.

Among the multiple molecules involved in immune system dysfunction in obese individuals, increased production of leptin in obese patients has an important role in enhancing the pro-inflammatory phenotypes of leukocytes. Increased concentrations of leptin promote production of pro-inflammatory cytokines, activation of Th1 responses and NK cells and reduced proliferation and differentiation of Treg cells [151]. High salt content in western-type diets has also been related to susceptibility to autoimmune and cardiovascular pathologies by activation of Th17 responses in animal models [152], although these effects have not been definitively established in humans. More specific detrimental effects of western-type diets have been determined for induction of oxidative stress and inflammation in the intestine, promoting dysbiosis of the intestinal microbiome and development of colorectal cancer [153].

As mentioned previously, traditional Mediterranean diets are considered beneficial for human health due to their putative anti-inflammatory and anti-oxidant properties. Studies in humans have demonstrated sex-specific changes in the expression of CD40 and CD86 co-stimulatory molecules after in vitro stimulation of peripheral blood leukocytes from female participants consuming a Mediterranean-style diet and vitamin D supplements for one year, although no significant differences were observed between patients on the Mediterranean diet and patients on a control diet in other immunological parameters, including leukocyte proliferation, T cell cytotoxic activity or cytokine production [154]. The monounsaturated oleic acid, found as a main constituent in olive oil, possesses anti-inflammatory characteristics, decreasing production of IL-1β, IL-6, and macrophage and neutrophil pro-inflammatory activity [155]. In addition, the phenolic compounds found in components of the Mediterranean diet, like olives, olive oil, and red wine, have anti-inflammatory and anti-oxidant properties, inhibiting activation of NF-κB, JNK, and STAT3 signaling pathways and reducing the expression of cyclooxygenase-2 and inducible nitric oxide synthase enzymes, as well as the production of IL-1β, IL-6, TNF-α, and CCL1 in patients with arthritis [26].

Finally, the beneficial health properties of Asian diets depend on the presence of relatively high concentrations of bioactive substances like quercetin, a flavonol that is present in fruits and vegetables, including apples, onions, shallots, tomatoes, asparagus, grapes, nuts, berries, and plant barks and leaves [156]. Quercetin inhibits production of pro-inflammatory cytokines, including IL-1 and TNF-α by macrophages and glial cells, as well as the expression of cyclooxygenase and lipoxygenase enzymes, mast cell degranulation and expression of adhesion and costimulatory molecules by different cell types [157]. It also enhances secretion of adiponectin and inhibits production of nitric oxide and TNF-α in adipose tissue in obese rats and decreases the severity of autoimmune disease by interfering with the activation of Th1 and Th17 responses by inhibiting the production of IFN-γ and IL-17 in animal models [157].

As with the microbiome, some dietary components have direct influences on the activation and evolution of different types of immune responses. The specific effects of some of these components are described in the following section.

## 6. Regulation of the Immune and Nervous Systems by Dietary Components and Their Implication in Neurological Disease

**Figure 2:**
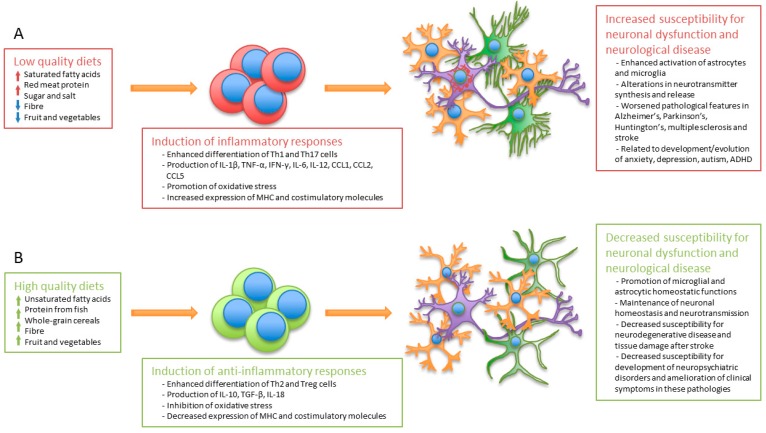
Dietary patterns determine the susceptibility to develop immune dysfunction and neurological abnormalities. Diverse components in diet modulate the differentiation and activation of specific leukocyte subpopulations, promoting pro- or anti-inflammatory functions. The effector functions of activated leukocytes regulate in turn the susceptibility to develop CNS inflammation, metabolic dysregulation, and tissue damage and are, thus, related to the development of neurological and psychiatric disorders. The figure depicts the effects of (**A**) low quality (e.g., western-type diets) and (**B**) high quality (e.g., Mediterranean diets) diets on the immune system and their relationship to the susceptibility for neuronal dysfunction and development of neurological and psychiatric disorders. MHC: Major histocompatibility complex.

The quality of diet is relevant for the regulation of the immune system, since multiple dietary components or their metabolic products possess direct immunomodulatory properties. Similarly, these components may also have direct or indirect effects on neurological functions, thus linking diet to immune regulation, neuronal homeostasis, and mental health (Figure 2).

### 6.1. Long-Chain Fatty Acids (LCFAs)

Long-chain fatty acids, such as the well-known omega-3 and omega-6 varieties, constitute essential components of cell membranes and cellular energy metabolism. Omega-6 LCFAs include arachidonic, linolenic, and linoleic acids, whereas omega-3 LCFAs include eicosapetaenoic (EPA) and docosahexaenoic (DHA) acids. Both types of LCFAs are essential and must be obtained from dietary components [158]. Similar to SCFAs, LCFAs activate multiple intracellular signaling pathways by interacting with GPRs, particularly GPR40 and GPR120 [159]. Signaling through GPR40 has been shown to inhibit activation of NF-κB and expression of TNF receptor 2 [160].

There are multiple reports on the effects of omega-3 and -6 LCFAs on the immune response. Dietary supplementation with omega-3 LCFAs has been shown to inhibit the release of pro-inflammatory cytokines such as IL-1β, IL-6, and TNF-α from activated leukocytes, as well as decreasing production of the lipid pro-inflammatory mediators prostaglandin E2 and leukotriene B4 [161,162]. The mechanism through which omega-3 and -6 LCFAs interfere with inflammatory responses is thought to involve in part the competition of these molecules with arachidonic acid to produce multiple bioactive lipid components, since all of these employ similar enzymatic processes involving cyclooxygenase, lipooxygenase and cytochrome P450 enzymes [162]. In addition, omega-3 and -6 LCFAs are substrates for the production of various anti-inflammatory lipid mediators of the resolvin (Rv) and protectin (P) families. RvD1, RvD2, and PD1, derived from DHA, as well as RvE1, derived from EPA, are known to inhibit multiple leukocyte functions, including chemotaxis and tissue infiltration of polymorphonuclear cells, as well as production of IL-1β, IL-6, IL-23, and TNF-α [163]. Furthermore, DHA is also a precursor for the production of anti-inflammatory maresins, which are produced during the resolution phase of inflammation and whose activities also include inhibition of neutrophil chemotaxis and stimulation of macrophage removal of apoptotic cells, along with the reduction of leukotriene B4 synthesis and analgesic properties [163].

Contrary to SCFAs, LCFAs are implicated in the development of autoimmune CNS disease due to their ability to promote proliferation and differentiation of pathogenic Th1 and Th17 cells, as well as enhancing the production of IFN-γ, IL-2, IL-6, and IL-17 in EAE models [164,165]. Additionally, 12 h exposure of Schwann cells to LCFAs in culture promotes oxidative stress and mitochondrial dysfunction in these cells, suggesting a possible toxic mechanism related to alterations in LCFA oxidation in pathologies like diabetic neuropathy [166].

### 6.2. Vitamins

#### 6.2.1. Vitamin Effects on the Immune System

Vitamins act as modulators for a variety of immune system functions, including activation, proliferation and cytokine production by T cells. Dietary vitamins also play important roles in the modulation of the intestinal microbiome, since bacteria are sensitive to these components [167]. For example, vitamin A plays an important role in shaping the composition of the intestinal microbiome, with some bacterial genera being directly sensitive to intestinal retinol. Vitamin A deficiency affects SCFA production by intestinal bacteria and promotes alterations in the metabolism of glucose, lipids, amino acids, and nucleic acids. Importantly, vitamin A deficiency is associated with changes in bile acid metabolism and liver dysfunction [168,169].

Vitamin A can be used by intestinal dendritic cells to produce retinoic acid, an important factor in the activation of the mucosal immune system, enhancing the production of chemokines, adhesion molecules, and specific cytokines, such as TGF-β and IL-17, as well as promoting the differentiation and activation of both intestinal CD4+ Th17 effector cells and CD4+ FoxP3+ Treg cells, along with IgA-producing plasma cells [170,171,172]. The presence of intestinal Treg cells is relevant for the establishment of tolerance to ingested antigens, preventing the development of Th2-dependent allergic reactions to dietary components [173], although it has also been shown that retinoic acid promotes Th2 responses by enhancing production of IL-4, IL-5, and IL-13, while reducing production of IL-12, IFN-γ, IL-2, and TNF-α [174]. Retinoic acid promotes dendritic cell maturation and survival, enhancing expression of MHC-II and costimulatory molecules [175]. In contrast to its Th2 and Treg polarizing capacity, vitamin A-derived retinoic acid has been shown to enhance the proliferation and effector functions of cytotoxic CD8+ T cells in animal cancer models [176].

Similarly, the B group vitamins are essential for immune system homeostasis and functions. Thiamine (vitamin B_1_) has been shown to promote accumulation of naïve B cells in intestinal Peyer patches and to induce IgA production in the intestinal mucosa [177], as well as regulating leukocyte activation, proliferation, and production of pro-inflammatory cytokines upon antigenic stimulation [178,179]. Riboflavin (vitamin B_2_) deficiency has a negative effect on macrophage cell lines, decreasing cell adhesion, viability, production of reactive oxygen species, and phagocytic activity in vitro [180]. Similarly, acute riboflavin depletion or deletion of riboflavin kinase, the enzyme responsible for the production of flavin co-factors, in murine macrophages, prevents TNF receptor 1-dependent activation of NADPH oxidase and production of reactive oxygen species, decreasing macrophage production of nitric oxide, IL-1β, IL-10, and CCL2, and increasing susceptibility to bacterial infection [181,182]. Metabolism of riboflavin by bacteria is also relevant for the maintenance of effector- and memory-phenotype mucosal-associated invariant T cells, responsible for the rapid activation of pro-inflammatory and cytotoxic responses in reaction to infection in mucosal epithelium, lymph nodes, and spleen [183]. Importantly, the pathological alterations in the immune response observed in riboflavin deficiency can be quickly reversed by riboflavin supplementation.

Supplementation with niacin (vitamin B_3_) presents anti-inflammatory properties. It prevents translocation of NF-κB to the nucleus, inhibiting production of TNF-α, IL-1β, IL-6, and CCL2, as well as expression of adhesion molecules and chemotaxis in antigen-stimulated macrophage cell lines, an effect mediated by GPR109A, which also acts as a receptor for butyrate. In addition, GPR109A-dependent stimulation of colonic macrophages and dendritic cells promotes production of IL-10 and IL-18 and proliferation of Treg cells, reducing intestinal inflammation [184]. Pyridoxal phosphate (vitamin B_6_) is a coenzyme necessary for amino acid metabolism, among many other functions as a regulator of carbohydrate, lipid and heme metabolism. It has been demonstrated to promote anti-inflammatory and anti-oxidant effects, inhibiting activation of NF-κB and JNK and NLRP3-dependent production of IL-1β and IL18, as well as reducing production of reactive oxygen species by antigen-stimulated macrophages [185]. Furthermore, diet-induced B_6_-deficient mice show decreased T cell proliferation, differentiation, and IL-2 production, with enhanced IL-4 secretion after mitogenic stimulation [186]. Biotin (vitamin B_7_) deficiency has been linked to intestinal inflammation and neurological alterations. Biotin deficiency has multiple effects on the immune system. It has been shown to inhibit the proliferation and maturation of B and T lymphocytes, decreasing antibody production by B cells and cytotoxic activity by NK and T cells [187,188]. In contrast, it promotes NF-κB activity and enhances DC secretion of IL-1β, IL-12, IL-17, IL-23, TNF-α, and IFN-γ after antigenic stimulation [188]. These contrasting activities have been related to immune dysfunction and systemic metabolic alterations in biotin-deficient animals.

Folate (vitamin B_9_) interacts with vitamins B_6_ and B_12_, as well as homocysteine, in trans-methylation metabolic pathways that regulate various cell functions, including the response to oxidative stress [189]. It has been shown to regulate pro-inflammatory and effector functions in leukocytes. Increasing folate concentrations inhibits the cytotoxic capacity of NK cells and IL-10 production by mouse splenocytes, and it also reduces expression of IL-1β, TNF-α, CCL2, and CD40 in macrophage cell lines, suggesting that adequate folate concentrations are necessary to promote proper pro-inflammatory effector functions by leukocytes both in vivo and in vitro [190,191]. Finally, the effects of cobalamine (vitamin B_12_) on the immune system have not been as extensively studied; however, there is evidence that demonstrates that vitamin B_12_ deficiency is associated with increased IL-6 production by peripheral blood mononuclear cells in Alzheimer disease patients and with altered numbers of Treg cells in circulation in vitamin B_12_-deficient patients [192,193].

On the other hand, vitamin C, also known as ascorbic acid, is essential for the regulation of cellular metabolism, as it possesses significant antioxidant activity and acts as a cofactor for cellular functions that promote the maintenance of the extracellular matrix and epithelial barriers. Vitamin C deficiency is well known to cause immune system dysfunction and increased susceptibility to infections, since it participates in the regulation of multiple functions in the immune system. It has been shown to promote chemotaxis, phagocytic activity, and bacterial killing of neutrophils and macrophages, as well as lymphocyte proliferation and differentiation, production of antibodies by B cells and cytotoxic activity of NK cells [194,195,196]. Vitamin C also enhances the differentiation of Treg cells and modulates production of TNF-α, IL-1β, IL-6, IFN-γ, IL-10, and type I interferons [197,198,199]. Similar to vitamin C, vitamin E has been reported to enhance lymphocyte proliferation, NK cell-dependent cytotoxicity, Th1 responses, antibody production, and secretion of cytokines like IL-1β and IL-2 [200]. It also enhances the antibacterial activity of neutrophils and inhibits oxidative damage and apoptosis in macrophages [201,202].

Vitamin D (calcidiol, calcitriol) regulates a great variety of functions within the organism. In the intestine, it is involved in maintenance of epithelial homeostasis and absorption of phosphate and calcium, as well as proper development and functions of the immune system [203]. Although the evidence is limited, it appears that vitamin D also plays a role in the modulation of microbiome composition, with vitamin D-deficient mice having increased presence of Bacteroidetes in the intestine [204]. Vitamin D is involved in immune regulation of inflammatory bowel disease [205]. Vitamin D deficiency inhibits the differentiation of dendritic cells towards a tolerogenic phenotype, resulting in increased pathology in animal models of colitis [206,207]. Enhanced intestinal pathology is dependent on increased production of the pro-inflammatory cytokines IL-1β, TNF-α, IL-12, and IFN-γ, along with IL-10 and CCL1, in vitamin D receptor-deficient mice [205]. Vitamin D promotes the differentiation of tolerogenic dendritic cells by decreasing their expression of MHC-II and coestimulatory molecules, as well as decreasing production of IL-12 and increasing production of IL-10 [206]. In addition, vitamin D-dependent signaling enhances TCR-dependent activation of T cells, since antigen recognition through the TCR promotes cell surface expression of the vitamin D receptor, which up-regulates T cell intracellular signaling and activation [208], while it promotes differentiation of Th2 and Treg cells over Th1 and Th17 cells by decreasing production of IL-2, IFN-γ, IL-6, and IL-23 and enhancing production of IL-4 [207,209,210]. Finally, vitamin D inhibits proliferation of B cells and their differentiation towards IgG-producing plasma cells [211].

To conclude, vitamin K has recently been proposed to promote anti-inflammatory effects through the activation of the vitamin K-dependent Gla-rich protein (GRP), whose expression is upregulated after antigenic challenge and downregulates the activity of nuclear factor NF-κB, as well as inhibiting the expression of IL-1β and TNF-α in macrophage cell lines [212]. Decreased plasma concentrations of vitamin K_1_ have been described in patients with T2DM and its concentration appears to have an inverse correlation with glucose and lipid metabolism, insulin resistance, and pro-inflammatory activity [212,213]. On the other hand, vitamin K_2_ has been shown to suppress proliferation and promote IL-4 production in circulating leukocytes from healthy donors in a dose-dependent manner, an effect not observed for vitamin K_1_ [214].

#### 6.2.2. Vitamin Effects on the CNS

Vitamins are also essential for the maintenance of neuronal functions and CNS homeostasis. Vitamin A is involved in the promotion of neurogenesis and synaptic plasticity in the hippocampus, hypothalamus and olfactory bulb. It has been involved in the pathogenesis of neurodegenerative disease, like multiple sclerosis, where it is considered to play an important role in the regulation of the Th1/Th17/Treg balance, inhibiting inflammation and promoting neuronal survival [215]. Vitamin A deficiency is also implicated in worsening of age-related cognitive deficits in elderly people and in animal models of AD, which can be improved by vitamin A supplementation [216]. Interestingly, retinoic acid has been implicated in development of schizophrenia, since it is involved in modulation of neural wave patterns, attention, sleep, and circadian rhythms; furthermore, recent evidence suggests that genetic alterations in retinoic acid receptor-dependent signaling are responsible in part for decreased grey matter volume in the brain and incidence of severe cognitive deficits in humans [217].

The B group vitamins have multiple functions in the CNS. Thiamine is essential for the synthesis of myelin sheaths and preservation of axonal integrity in the nervous system [218]. Thiamine deficiency is related to development of neurological alterations in both the peripheral and central nervous systems observed in pathologies like Beriberi and Wernicke-Korsakoff syndrome, which respond rapidly to thiamine supplementation [219]. Although the molecular mechanisms relating thiamine to neurological symptoms have not been a focus of intense research, studies in animal models of neuroinflammation have demonstrated the role of thiamine in controlling leukocyte activation and effector functions. In EAE models, thiamine deficiency promotes CCL2 and CCR2 expression in the spinal cord, enhancing microglial activation and leukocyte infiltration in the central nervous system, favoring both Th1 and Th17 responses and stimulating lymph node- and spleen-derived T cell migration and proliferation in vitro [173]. Data on enhanced microglial activation during thiamine deficiency is supported by reports showing that a synthetic thiamine derivative (benfotiamine) inhibits activation of BV-2 microglia cells after antigenic stimulation by suppressing nuclear translocation of NF-κB and decreasing production of nitric oxide and the pro-inflammatory cytokines TNF-α and IL-6, while increasing production of IL-10 [174]. Thiamine has also been implicated in the regulation of stress responses, anxiety, depression and cognitive deficits in elderly people. Thiamine supplementation can ameliorate these disorders both in animal models and in humans, demonstrating beneficial effects that can be maintained long-term with regular vitamin supplementation [220,221].

Riboflavin is also considered an important participant in myelination, since its deficiency inhibits brain-derived neurotrophic factor (BDNF) production in the brain and promotes glutathione-dependent oxidative stress and peripheral nerve demyelination. It has also been reported that dietary supplementation with this vitamin improves disease characteristics in multiple sclerosis patients [222]. Niacin’s effects in the CNS are usually considered in combination with those from tryptophan and its metabolites, like serotonin and the kynurenines, as their metabolism is intrinsically interlinked. Therefore, it affects neurogenesis and neuronal survival, astrocyte homeostasis, neurotransmitter synthesis, oxidative stress, and vascular permeability at the blood-brain barrier. It has been implicated in development and evolution of age-related cognitive deficits and AD, as well as in inflammatory and oxidative damage in PD and HD, and traumatic and ischemic CNS injury [223]. As it is related to tryptophan metabolism, niacin has also been related to the incidence of psychiatric disorders like anxiety, depression and schizophrenia, via alterations in brain kynurenine concentrations [223]. Vitamin B_6_ is essential for neurotransmitter synthesis and for the conversion of glutamate to GABA. It has been used for successful, long-term treatment of drug-resistant epileptic seizures in some patients [224]. Deficiency of this vitamin has been implicated in myelin degeneration in animal models [225]. There is very limited information available on the possible effects of biotin for the modulation of neurological pathologies; however, it has been suggested that biotin may be a useful adjuvant for treatment of multiple sclerosis, due to its role in enhancing lipid synthesis and potentially promoting remyelination within the damaged CNS [226].

Similarly, previous studies have demonstrated that folate deficiency is involved in alterations of neural tube development and hippocampal functions relating to learning and memory deficits, probably stemming from neuronal toxicity and oxidative damage derived from increased homocysteine concentrations [227]. More importantly, folate deficiency is implicated in the pathogenesis of autism and schizophrenia. Research suggests that the presence of anti-folate receptor-alpha antibodies in circulation in early stages of development affects the development and normal functions of the brain, promoting the appearance of psychiatric disorders in humans [228]. Finally, cobalamin is an essential vitamin that can be obtained from fish, red meat, eggs, and milk. It is relevant for erythropoiesis, DNA synthesis, and normal CNS myelination. In CNS pathologies, cobalamin is considered important for the reduction of homocysteine-dependent oxidative stress and regulation of DNA synthesis and methylation. In particular, cobalamin deficiency is related to the development of subacute combined degeneration, which is characterized by myelin degeneration and TNF-α-dependent CNS inflammation, accompanied by reduced concentrations of IL-6 and epidermal growth factor, which are considered neuroprotective [229]. Cobalamin, along with folate and pyridoxal phosphate, has been proposed to have beneficial effects for the prevention of age-related cognitive decline and the evolution of AD and PD.

Regarding other vitamins, vitamin C is present in high concentrations within the CNS and it participates in a variety of processes in nervous tissue, including antioxidant and anti-inflammatory functions, promotion of neuronal proliferation and survival, oligodendrogenesis, enhancement of myelin formation, and regulation of neurotransmitter synthesis and release, particularly for epinephrine, norepinephrine, and dopamine, and the regulation of the permeability of the blood-brain barrier and glucose and lactate metabolism at neuronal synapses [230]. Vitamin C deficiency enhances amyloid plaque formation, blood-brain barrier dysfunction, oxidative stress and neuronal damage in animal models of AD, as well as oxidative damage in models of ischemic CNS injury. Vitamin C also protects neurons against glutamate-dependent excitotoxic damage and promotes the differentiation of dopaminergic neurons in vitro, while lower plasma concentrations of vitamin C have been found in patients with PD and multiple sclerosis [230]. According to its antioxidant and neuroprotective functions, vitamin C demonstrates anti-depressant and anxiolytic effects in multiple studies, mainly through modulation of serotonin- glutamate- and GABA-dependent neurotransmission. Patients with anxiety or depression present decreased plasma vitamin C concentrations compared with healthy subjects and its supplementation has a beneficial effects on clinical symptoms. Deficiency of this vitamin may also enhance the severity of disease in patients with schizophrenia [230].

The effects of vitamin D have been studied mostly in the context of neuroinflammation and demyelination, with several studies addressing the potential benefits for vitamin D supplementation in patients with multiple sclerosis; however, no clear-cut effects or mechanisms of action have been determined [231]. It appears that the suggested protective mechanisms relate mostly to the immunomodulatory properties of vitamin D, rather than to direct neuroprotective effects. Vitamin D supplementation may inhibit CNS traffic of leukocytes and differentiation of Th17 cells, while also enhancing production of TGF-β, thus decreasing systemic inflammatory responses, which would have beneficial effects for the patients [207]. Vitamin D has also been related to the development and severity of depression and attention deficit-hyperactivity (ADHD) disorder. It has been suggested that it regulates emotion and behavior, since the prefrontal cortex, basal ganglia, and hypothalamus express receptors for this vitamin and these areas are related to the control of emotions and behavior. Similarly, vitamin D deficiency appears to be related to decreased production of serotonin, one of the main targets in depression [231,232]. On the other hand, vitamin D concentrations are lower in children with ADHD and a six-week vitamin D supplementation regime has been reported to improve clinical symptoms in these patients, particularly with respect to inattention [233].

The beneficial effects of vitamin E in the CNS have been mostly related to its antioxidant properties. Deficiency of this vitamin promotes CNS oxidative damage, particularly in Purkinje neurons in the cerebellum, and is related to development of spinocerebellar ataxia, which can be prevented by vitamin E supplementation in animal models [234]. Vitamin E also demonstrates neuroprotective properties in models of spinal cord injury, where it promotes neuronal survival and oligodendrocyte proliferation and reduces oxidative damage, and improving motor function and recovery after tissue damage [235]. Vitamin E supplementation also improves neurotransmission in brain slices in animal models of PD [236]. Regarding mental health, vitamin E deficiency is related to increased glutamate levels and development of anxiety, which may be prevented by vitamin supplementation in animal models [237]. Finally, plasma concentrations of vitamin E are related to cognitive performance in older humans, where lower concentrations of this vitamin in circulation correlate with increased markers of systemic oxidative damage and decreased cognitive score in a longitudinal study of cognitive impairment in older adults [238].

Finally, vitamin K improves anxiety- and depressive-like behavior in animal models of metabolic syndrome, although the mechanism involved was suggested to depend on an indirect effect through modulation of glucose metabolism [239]. Increased concentrations of vitamin K are related with improved cognitive performance in humans, and using vitamin K antagonists induces cognitive alterations in executive functions in geriatric patients [240], therefore, demonstrating the role of vitamin K in proper neuronal functioning, although the precise mechanisms involved in these effects have not been determined.

### 6.3. Amino Acids

Like vitamins, amino acids are essential cellular components required not only for protein synthesis, but also as substrates for a multitude of metabolic functions regulating the immune and nervous systems. Arginine is an important amino acid for the regulation of macrophage and lymphocyte effector functions. Arginine is required for nitric oxide production in activated phagocytes and arginase expression in macrophages is related to decreased inflammation in models of inflammatory disease [241]. Similarly, arginine modulates T cell proliferation [242]. Arginine may be transformed by intestinal bacteria into metabolites denominated polyamines, including spermine, spermidine and putrescine. Intestinal arginine concentrations are higher in germ-free mice, suggesting that the microbiome has a significant role in intestinal arginine metabolism [243,244]. These compounds are known to promote intestinal homeostasis and maturation of intestinal intraepithelial leukocytes, including CD4+ and CD8+ T cells, B cells and NK cells [245]. Spermine also inhibits antigen-dependent production of pro-inflammatory cytokines by macrophages, promoting anti-inflammatory effects in various models [246,247]. In the CNS, arginine is involved in the regulation of oxidative stress and neurological alterations in neuroinflammatory, neurodegenerative, and psychiatric disorders, including stroke, AD, PD, epilepsy, autism, obsessive-compulsive disorder, depression, anxiety, and schizophrenia [248,249,250]. Agmantine, a metabolite derived from arginine decarboxylation that is found throughout the brain and particularly in the hypothalamus, is capable of selectively blocking neurotransmission through NMDA receptors in specific areas of the brain, like the hippocampus, and it has been shown to attenuate nitric oxide production, oxidative stress, and glutamate-dependent excitotoxicity in animal models of dyskinesia and hyperalgesia [250]. Furthermore, intra-peritoneal supplementation with agmantine reduces neuronal damage, atrogliosis, lipid peroxidation and production of IL-1β and TNF-α in animal models of PD [251]. Additionally, there is evidence that agmantine may have anti-depressant, anxiolytic, and anti-convulsive properties, derived from its capacity to bind to alpha-2 adrenergic receptors, nicotinic and muscarinic acetylcholine receptors, as well as alpha-amino-3-hydroxy-5-methylisoxazole-4-propionic acid (AMPA) receptors [250]. Finally, the use of a D- analog of arginine decreases arginine-dependent oxidative damage and neurotoxicity in the CNS [248]. Therefore, arginine is an important metabolite for the maintenance of neuronal homeostasis.

Similar to arginine, tryptophan has important immunoregulatory properties. Tryptophan is required for T cell proliferation and survival after activation [252], and its metabolism through the enzyme IDO promotes different effects on leukocytes, including dendritic-cell-dependent suppression of T cell responses [253]. Excessive consumption of dietary tryptophan is related to alterations in IDO activation and changes in eosinophil traffic and inflammatory activity in humans [254]. As mentioned previously (see AhR section), animal models of neuroinflammation, such as EAE, have demonstrated that tryptophan is important for the evolution and severity of pathology and that its effect is dependent on AhR, since activation of this receptor decreases disease severity by promoting differentiation of T cells to a regulatory phenotype, instead of the pro-inflammatory Th17 subpopulation and administration of dietary tryptophan modulates disease evolution in wild-type, but not in AhR-knockout animals [255]. Elimination of the intestinal microbiome by administration of ampicillin increases the severity of disease and this effect can be rescued by supplementation of bacterial enzymes for tryptophan metabolism or by direct administration of tryptophan metabolites of bacterial origin. Modulation of disease in this animal model appears to be dependent on regulation of microglial activation through the AhR, with microglia in turn regulating astrocyte activation and production of pro-inflammatory factors within the CNS [256]. Concentrations of AhR in plasma are decreased in patients with multiple sclerosis, with AhR activity changing in response to disease activity [112].

Taurine may be encountered in the intestine as a byproduct of bacterial deconjugation of secondary bile acids produced in the liver. It has been demonstrated that taurine enhances NLRP6-dependent production of IL-18 by intestinal epithelial cells, which is important for the maintenance of epithelial homeostasis by enhancing cell proliferation, mucus secretion, and production of antimicrobial peptides. The relevance of the microbiome for IL-18 production in the intestine has been demonstrated by showing decreased inflammasome activity and IL-18 concentrations in germ-free mice [257]. Taurine is present in high concentrations in the CNS, where it regulates ion and fluid exchange, as well as neurotransmission. It also acts as an agonist of glycine and GABA_A_ receptors and, thus, it has been considered to have a potential role in neurological diseases associated to dysregulation in CNS inhibitory signals, like epilepsy and depression [258]. The therapeutic utility of taurine supplementation for prevention or treatment of neurological disease has been demonstrated in studies of high-dose supplementation with taurine in animal models of intracerebral hemorrhage, showing that this regime decreases edema and tissue damage, reducing leukocyte infiltration into the CNS, gliosis, and production of pro-inflammatory cytokines [259]. Similarly, taurine supplementation before disease induction in paraquat-dependent Parkinson’s-like disease in mice inhibits NF-κB-dependent microglial activation and oxidative stress, decreasing neurotoxicity [260].

### 6.4. Polyphenols

The polyphenols include multiple compounds derived from plants, such as the flavonoid catechins found in different varieties of green, black and oolong teas, and the non-flavonoids curcumin, from the *Curcuma longa* plant, and resveratrol in grapes and berries. All of these compounds share similar properties, decreasing adipocyte proliferation and maturation, inhibiting lipogenesis and promoting lipolysis [261,262,263,264]. They also share similar anti-inflammatory properties. Studies of green tea supplementation in animal models have shown that polyphenols from green, black, and oolong teas are related to decreased production of IL-1β, IL-6, and CCL1, as well as increasing expression of antioxidant superoxide dismutase and anti-inflammatory adiponectin in animals fed high-fat or high sucrose diets [265,266]. Curcumin also prevents NF-κB activation and release of CCL1, TNF-α, and IL-6 from adipocytes [267]. Similarly, resveratrol has demonstrated anti-inflammatory properties that include the inhibition of NF-κB activation and production of CCL1, TNF-α, and IL-6 by adipocytes and antigen-stimulated macrophages [268,269].

The metabolic transformation of polyphenols by the intestinal microbiome produces metabolites that interfere with amyloid beta aggregation in vitro [270] and polyphenols from grape seeds are also able to inhibit aggregation of Tau peptides [271], implying that these compounds may be beneficial for the prevention and treatment of neurodegenerative pathologies like AD and PD. This idea is backed up by evidence demonstrating that dietary supplementation with grape-derived polyphenols decreases disease severity and progression, maintaining cognitive performance in memory tests, in animal models of AD [272].

### 6.5. Minerals

Minerals are essential components of diet and are necessary for a large variety of functions within the organism. The functions of minerals in the immune system are too numerous to properly describe in this review, but a few examples include the functions of iron as an important component for antibacterial responses, since it is essential for cellular metabolism and redox reactions [273]. Copper is employed by phagocytes to induce bacterial toxicity [274]. Selenium is part of cellular glutathione-dependent antioxidant mechanisms and antimicrobial compounds in neutrophils, as well as for T cell and NK cell cytotoxic activity [275], while zinc deficiency affects thymic development, leukocyte proliferation, and T cell differentiation [276].

Likewise, minerals like iron, zinc, selenium, copper, manganese, and magnesium have all been implicated in CNS homeostasis and regulation of neuronal survival and functioning. For example, alterations in the copper/zinc ratio, iron and selenium concentrations have been observed in circulation in patients with depression and there are reports of alterations in the concentrations of iron, magnesium and zinc in ADHD [277,278]. Similarly, zinc supplementation has been suggested as a therapeutic alternative in depression and to prevent age-related cognitive deterioration [277]. Deficits in selenium are related to neurodegenerative pathologies like AD and PD, and this mineral has been proposed as a potential biomarker for disease development [279], while iron deficiency is widely known to affect CNS myelination and human cognition [280].

## 7. Diet as an Adjuvant for the Prevention of Neurological Disease and Maintenance of Mental Health

As described throughout this review, diet affects the composition and characteristics of the intestinal microbiome, as well as the functions of the immune and nervous systems, through multiple mechanisms. Chronic changes in dietary patterns are associated with a large variety of metabolic alterations, which may have important implications for neurological homeostasis and mental health.

Obesity, associated with high-energy content diets, promotes chronic, systemic low-grade inflammation affecting several metabolic pathways, including tryptophan processing via IDO, leading to production of kynurenines, an effect that has been extensively related to psychiatric pathologies like anxiety and depression [281]. Kynurenine and quinolinic acid, tryptophan metabolites produced by IDO activation, are known to possess potentially detrimental properties for the nervous system, as they enhance oxidative stress and neuronal apoptosis and potentiate anxiety- and depression-like behavior in animal models and in humans [282,283]. Interestingly, these types of behavior are usually elicited in animal models by antigenic stimulation with bacterial components like LPS, stimulating pro-inflammatory responses characterized by enhanced production of IL-β, TNF-α, and IL-6, both systemically and within the CNS, particularly in areas like the hippocampus and limbic system, that lead to alterations in emotional, behavioral and cognitive functions, particularly those related to learning and memory, by modifying neurotransmitter synthesis and serotonin-, glutamate-, and dopamine-dependent signaling [281,282,283]. Both anxiety- and depressive-like behavior are strongly related to diet composition, as consumption of western-type diets worsens these characteristics in experimental animal models of anxiety and depression. High-fat diets also promote long-term cognitive deficits in learning and memory that can be established during pregnancy or lactation in animal models and involve deficient production of BDNF and changes in intracellular signaling pathways related to insulin, which can be reversed in part by administration of beneficial dietary components, like resveratrol [284]. Similarly, high-fat diets promote CNS oxidative stress and inflammation and worsen disease in animal models of AD and traumatic brain injury, affecting the production of neurotrophic factors, the survival of neurons in the hippocampus and amygdala and promoting cognitive deterioration and emotional and behavioral alterations [285,286]. High-fat diet-dependent CNS inflammation and cognitive deficits can also be reverted in part by supplementation with resveratrol [287].

In contrast, Mediterranean diets have been associated to decreased risk for developing neurological and psychiatric pathologies, particularly for the incidence of stroke, depression and development of age-related cognitive impairment and neurodegeneration in AD and PD [288,289]. Some of the beneficial effects of the Mediterranean diet on development of neurological and psychiatric disorders have been attributed to increased consumption of fruit, vegetables, fish, olive oil, and high contents of omega-3 unsaturated fatty acids and resveratrol whereas, in general, poor diet quality is related to higher incidence and prevalence of these pathologies [290].

Supporting the idea of a direct relationship between the diet, the microbiome and neuronal homeostasis and mental health, recent studies have demonstrated that the microbiome plays a pivotal role in the regulation of leptin and insulin sensitivity through production of bioactive metabolites, which affect tryptophan metabolism, neurotransmitter synthesis, particularly for serotonin and dopamine, and production of neurotrophic factors, promoting anxiety- and depressive-like behaviors that can be ameliorated by antibiotic treatment in animal models [291]. Additionally, probiotic treatment with *Lactobacillus* and *Bifidobacterium* species reverses the negative changes associated to intake of high-fat diets, like increased intestinal permeability, LPS-dependent chronic low-grade inflammation and insulin- and leptin-resistance, which are also associated to development of anxiety and depression [292]. Multiple studies have related intestinal dysbiosis with increased susceptibility for a variety of neurological and psychiatric pathologies. For example, autism-spectrum disorders are related to increased quantities of *Clostridium* and Enterobacteriaceae and decreased presence of *Bifidobacterium* and *Akkermansia* species; depression is associated to the presence of Bacteroidetes and Proteobacteria and decreased *Bifidobacterium* and *Lactobacillus*; stress associates with increased presence of *Clostridium* and decreased *Bacteroides* and *Lactobacillus*; finally, AD and PD correlate with increased amounts of Bacteroidetes and Entereobacteriaceae, with decreased Actinobacteria, Firmicutes, Proteobacteria, and Prevotellaceae. Therefore, the use of probiotics has demonstrated an important therapeutic potential for the treatment of neurological and psychiatric disorders in animal models and in humans [292].

The relationship between the diet, microbiome and mental health depends on a large variety of stimuli, including dietary components, microbial metabolites, gastrointestinal, endocrine, neuronal, and immunologic signals, all of them acting in concert to modulate systemic metabolism and tissue functions. Consequently, dietary patterns influence not only the development of immunological responses, but also the homeostasis and functions of the CNS. Nonetheless, although altered immune functions are a recognized common characteristic in neurological pathologies, research on the beneficial effects of diet on the immune and nervous systems remains mostly separate. There is a great need for studies integrating the effects of diet on the three main components discussed herein, the microbiome, immune, and nervous systems, together, in order to better understand their interplay in the regulation of neuronal homeostasis and mental health.

## 8. Concluding Remarks

The profound effects of diet on the regulation of the intestinal microbiome, the immune system and the central nervous system make it a powerful tool for prevention and therapeutic intervention in a large range of human pathologies. In particular, the potential for therapeutic benefits derived from relatively simple dietary interventions in neurological and psychiatric pathologies must become a focus of more intensive research, since it opens up the possibility of ameliorating disease in patients suffering from emotional and behavioral disorders, like anxiety and depression, as well as in neurodegenerative pathologies and age-related cognitive decline. The evidence condensed in this review demonstrates the need for a balanced, high-quality diet to promote immune and neuronal homeostasis in order to maintain mental health.
